# Zoonoses and emerging pathogens

**DOI:** 10.1186/s12866-023-02984-w

**Published:** 2023-08-23

**Authors:** Krzysztof Skowron, Katarzyna Grudlewska-Buda, Faham Khamesipour

**Affiliations:** 1 Department of Microbiology, Ludwik Rydygier Collegium Medicum in Bydgoszcz, Nicolaus Copernicus University in Toruń, Toruń, Poland; 2 Research Center for Hydatid Disease in Iran, Kerman University of Medical Sciences, Kerman, Iran

**Keywords:** Zoonoses, Emerging pathogens, Virulence factors, Virus, Bacteria, Protozoa, Fungi

## Abstract

Zoonoses represent a major challenge for many disciplines, including microbiology, epidemiology, veterinary, medicine and ecology. Moreover, they pose severe risks to human health and economy. In this editorial, we invite contributions to a *BMC Microbiology* collection on ‘Zoonoses and emerging pathogens’, covering research on the pathogenesis, identification, treatment and control of zoonoses.

Some of the qualities that define modern times are the speed and the magnitude at which change occurs. We live in an increasingly globalized world, characterized for instance by international exchanges and economic trades, and land-use changes. Most of these anthropogenic factors may have an impact in the diffusion of zoonoses and pathogens, and consequently human health [1].

Nowadays, many research areas and disciplines, including microbiology, face new challenges related to the rapid identification and control of pathogens, as well as understanding the mechanisms underlying their spreading. Currently, zoonotic diseases are a significant, widespread issue with potential major implications for the global health of our society [2].

The majority of human infectious diseases have animal origins [3]. These pathogens not only cause animal diseases, but could also pose a severe threat to human health [4]. Zoonotic diseases are defined as infectious diseases in which the source of pathogens is either an animal or its secretions, or food contaminated with microorganisms (e.g. eggs and meat) or the environment (e.g. soil and plants) (World Health Organization, ‘Zoonoses’) [1]. Zoonoses comprise both existing and newly-identified infectious diseases (‘emerging infectious diseases’, EIDs) [1]. EIDs are zoonotic diseases that are either newly diagnosed, or have occurred previously but currently show an increase in incidence or range of geographic, vector, or host expansion. It is estimated that about 60% of EIDs are related to the transmission of microorganisms from animals to humans [5]. With over 200 known types of zoonoses, zoonotic diseases can be caused by viruses, bacteria, fungi, as well as protozoa and worms [6]. Nowadays, zoonotic diseases can have an unprecedented and global impact on the human population, due to their diversity, spreading capacity and severity. The list of major zoonotic viral diseases and pathogens includes coronaviruses (e.g. the Middle East respiratory syndrome (MERS) and severe acute respiratory syndrome (SARS)), flaviviruses (e.g. the yellow fever and West Nile virus), Crimean-Congo hemorrhagic fever, hemorrhagic fever viruses (e.g. Ebola and Marburg), rabies and influenza viruses [7, 8]. The most common and concerning zoonotic bacterial diseases currently include the plague, Q fever, salmonellosis, listeriosis, zoonotic tuberculosis, anthrax, brucellosis, leptospirosis, campylobacteriosis, psittacosis, while widespread parasitic zoonoses comprise e.g. toxoplasmosis, cysticercosis, echinococcosis, cryptosporidiosis, giardiasis [7, 8].

Over the years, many epidemics and pandemics have been caused by zoonoses, including the very recent pandemic due to the coronavirus, SARS-CoV-2 (COVID-19) [1, 9]. Outbreaks of zoonoses can be associated with high potential for mortality and represent a severe threat to public health, possibly causing major financial losses in the economic and commercial sectors. To reduce the risk of epidemics and pandemics, monitoring and studying the emergence of zoonotic pathogens is critical for our society [10]. The severity and impact of zoonotic diseases depend on many environmental, economic and social factors, including the climate, geographic location, population density and sanitation, with zoonoses affecting both rural and urban areas [1, 11]. Zoonotic disease can be transmitted in different ways, either directly (by contact with body fluids of infected animals, such as saliva, blood, mucous, and feces, or through bites and scratches) or indirectly (by contact with animal habitats or vectors, such as insects). Alternatively, consuming food (e.g. milk, meat, eggs) contaminated with feces from farm animals can represent a source of zoonoses [12]. Traditional food involving the consumption of raw meat (from wild animals) or traditional medicine can also contribute to the occurrence of infectious zoonotic diseases [13]. In addition, the current and global climate crisis can further enhance the transmission of zoonotic diseases among the world population, as reported for several vector-borne diseases, and mainly in tropical low- and middle-income countries [1, 10–12]. In recent years, changes in the timing and magnitude of temperature and rainfall have also accelerated the spreading of infectious diseases in northern countries [1].

‘One Health’ is an integrated program established by the World Health Organization that aims to unify and optimize the health of humans, animals and the environment [3, 9]. Some of the essential pillars of the ‘One Health’ approach are disease prevention, control and management, aiming to contribute to global health security. In this context, developing quick and correct diagnostic methods is essential to monitor and control emerging pathogens and zoonoses, as the misidentification of pathogens can result in inadequate treatment or inability to prevent disease transmission and spreading [5, 6].

Currently, we have poor understanding of why some zoonoses arise and spread more efficiently than others, what are the mechanisms underlying their success, as well as what strategies could be adopted to control them. Moreover, knowledge on EIDs, especially in the context of newly emerging (viral, parasitic, bacterial and fungal) pathogens, remains limited. Important unresolved questions include, for instance, the identification of transmission routes for EIDs and achieving a better understanding of recent epidemics/pandemics. Additionally, there is an urgent need to comprehend the increasing antimicrobial resistance, which affects both human and veterinary medicine. As both socioeconomic and environmental factors can affect and enhance the spreading of zoonotic diseases, studies addressing this research area are today more important and timelier than ever. Here, we encourage submissions to the *BMC Microbiology* collection ‘Zoonoses and emerging pathogens’, aiming to cover research on the pathogenesis, epidemiology, pathogen-host interactions, detection, diagnosis, treatment, control and prevention of zoonoses and emerging pathogens.

## Data Availability

Not applicable.

## Abbreviations

COVID-19: Coronavirus disease 2019
EIDs: Emerging infectious diseases
MERS: Middle East respiratory syndrome
SARS: Severe acute respiratory syndrome
